# Healthy Lifestyle Behaviors and Technostress: A Combined Lifestyle Score Analysis in 104,175 Spanish Workers

**DOI:** 10.3390/nu18172754

**Published:** 2026-08-23

**Authors:** Marta González Rivas, Ángel Arturo López-González, Diego González Carrasco, Carla Busquets-Cortés, Lluis Rodas Cañellas, José Ignacio Ramírez-Manent

**Affiliations:** 1 ADEMA University School, University of the Balearic Islands, 07009 Palma, Spain; 2 Primary Care, Balearic Islands Health Service, 07010 Palma, Spain

**Keywords:** technostress, healthy lifestyle score, physical activity, Mediterranean diet, occupational health, digital well-being

## Abstract

**Background:** Technostress has emerged as a major occupational health concern in increasingly digitalized workplaces. Although organizational and technological determinants of technostress have been widely investigated, the potential influence of healthy lifestyle behaviors remains poorly understood. This study aimed to evaluate the association between a combined Healthy Lifestyle Score (HLS) and technostress in a large cross-sectional sample of Spanish workers. **Methods:** A cross-sectional study was conducted among 104,175 workers who underwent routine occupational health examinations between January 2021 and December 2024. The Healthy Lifestyle Score was constructed by assigning one point each for regular physical activity, high adherence to the Mediterranean diet, and non-smoking status, resulting in scores ranging from 0 to 3. Technostress was assessed using the Technostress Short Questionnaire (TCS-Short) and categorized as low, moderate, high, or very high. Logistic regression models were used to estimate crude and adjusted odds ratios (ORs) and 95% confidence intervals (CIs) for high-to-very high technostress according to HLS categories. **Results:** Significant differences in technostress levels were observed across HLS categories (*p* < 0.001). The prevalence of high-to-very high technostress was 50.8% among workers with HLS = 0 and 50.7% among those with HLS = 1, decreasing markedly to 7.5% and 7.2% among workers with HLS = 2 and HLS = 3, respectively. After adjustment for sex, age, educational level, and social class, participants with HLS = 2 and HLS = 3 exhibited substantially lower odds of high technostress (OR = 0.073, 95% CI 0.068–0.078 and OR = 0.074, 95% CI 0.069–0.079, respectively) compared with those with HLS = 0. The categorical analysis suggested a threshold-like rather than progressive association, with substantially lower odds of high-to-very high technostress observed among workers with HLS = 2 and HLS = 3, whereas HLS = 1 did not show lower odds compared with HLS = 0. The final model demonstrated excellent discrimination (AUC = 0.902). **Conclusions:** Healthy lifestyle profiles were strongly associated with technostress in this large cross-sectional occupational sample. Workers with HLS = 2 and HLS = 3 showed markedly lower odds of high-to-very high technostress than those with HLS = 0, whereas HLS = 1 did not show lower odds. Component-specific analyses indicated that the associations differed substantially across the behaviors comprising the score, supporting interpretation of the HLS as an unweighted behavioral count rather than as a measure of equivalent contributions from each component.

## 1. Introduction

The digital transformation of modern workplaces has increased workers’ reliance on information and communication technologies (ICTs), improving productivity and flexibility while also generating new psychosocial demands. Among these, technostress has emerged as an important occupational health concern and is commonly understood as a form of stress arising when technology-related demands exceed an individual’s resources, skills, or coping capacities. It encompasses several dimensions, including techno-overload, techno-invasion, techno-complexity, techno-insecurity, and techno-uncertainty, and has been associated with burnout, emotional exhaustion, psychological distress, reduced job satisfaction, impaired work engagement, and poorer well-being [1,2,3,4]. Its relevance became particularly evident during and after the COVID-19 pandemic, when remote and hybrid work increased dependence on digital technologies and exposure to continuous connectivity, interruptions, information overload, and blurred work–life boundaries [5,6,7].

Although organizational and technological determinants of technostress have been widely investigated, considerably less attention has been paid to the potential role of individual health-related behaviors. This gap is particularly relevant because responses to psychosocial stressors are influenced not only by external demands but also by behavioral and physiological factors that determine resilience, recovery, and adaptation. Workers exposed to similar technological environments may experience substantially different levels of stress depending on their health status, lifestyle characteristics, and coping resources. Understanding these factors may provide valuable opportunities for prevention and intervention, particularly because many lifestyle behaviors are potentially modifiable.

Physical activity represents one of the most extensively studied protective factors against psychological stress. Regular exercise has been associated with lower perceived stress, improved mood, enhanced emotional regulation, better sleep quality, and greater psychological resilience. Multiple biological mechanisms may explain these effects, including modulation of hypothalamic–pituitary–adrenal axis activity, reductions in systemic inflammation, improvements in autonomic nervous system balance, and increased production of neurotrophic factors involved in brain plasticity [8,9,10]. In occupational settings, physically active workers generally report lower levels of stress and better mental health outcomes than sedentary individuals. These observations suggest that physical activity may also be associated with better adaptation to technology-related demands and lower vulnerability to technostress.

Dietary habits may constitute another important determinant of stress resilience. In recent years, increasing attention has been devoted to the relationship between nutrition and mental health, giving rise to the field of nutritional psychiatry. Among dietary patterns, the Mediterranean diet has consistently demonstrated beneficial associations with psychological well-being, depressive symptoms, cognitive performance, and quality of life [11,12,13]. Characterized by high consumption of fruits, vegetables, legumes, whole grains, olive oil, fish, and nuts, the Mediterranean dietary pattern exerts anti-inflammatory and antioxidant effects that may influence neurobiological pathways involved in stress regulation. Emerging evidence also suggests that diet may affect mental health through modulation of the gut–brain axis, metabolic homeostasis, and sleep quality. Although studies specifically evaluating Mediterranean diet adherence in relation to technostress remain scarce, these mechanisms provide a strong biological rationale for a potential protective association.

Smoking behavior is another lifestyle factor frequently linked to stress and mental health outcomes. Despite the widespread perception that smoking may alleviate stress, epidemiological evidence consistently indicates that smokers tend to report higher levels of perceived stress, anxiety, and psychological distress than non-smokers [14,15]. Nicotine dependence may create cyclical fluctuations in mood and physiological arousal that reinforce stress-related symptoms rather than alleviate them. Consequently, smoking is generally regarded as a marker of maladaptive coping in response to chronic psychosocial challenges. Evaluating smoking alongside physical activity and dietary habits may therefore contribute to a more comprehensive understanding of lifestyle-related vulnerability and resilience factors in the context of technostress.

Importantly, lifestyle behaviors rarely occur in isolation. Individuals who engage in regular physical activity are more likely to follow healthy dietary patterns and avoid smoking, whereas unhealthy behaviors frequently cluster within the same individuals. For this reason, contemporary public health research increasingly focuses on combined lifestyle scores rather than isolated behaviors. Healthy lifestyle indices integrate multiple health-related habits into a single measure that better reflects overall behavioral profiles and allows assessment of cumulative effects on health outcomes. Previous studies have demonstrated that higher healthy lifestyle scores are associated with lower risks of cardiovascular disease, type 2 diabetes, metabolic syndrome, non-alcoholic fatty liver disease, cancer, and all-cause mortality [16,17,18,19]. However, despite the growing interest in lifestyle clustering, virtually no large-scale occupational studies have examined whether a combination of healthy behaviors is associated with the likelihood of experiencing technostress.

Addressing this gap is particularly relevant because technostress may represent an intermediate mechanism linking unhealthy lifestyles, psychosocial stress, and adverse cardiometabolic outcomes. Chronic exposure to stressors has been associated with neuroendocrine dysregulation, sleep disturbances, inflammatory activation, unhealthy eating behaviors, physical inactivity, and increased cardiometabolic risk [20,21,22]. If healthy lifestyle behaviors are associated with greater resilience to technology-related stress, such behaviors may represent relevant targets for further investigation in increasingly digitalized workplaces.

Therefore, the aim of the present study was to evaluate the association between a combined Healthy Lifestyle Score and elevated technostress in a large cross-sectional sample of Spanish workers. The score was constructed using three modifiable health behaviors: regular physical activity, adherence to the Mediterranean diet, and non-smoking status. We hypothesized that workers with higher Healthy Lifestyle Scores would exhibit lower levels and odds of high technostress than those with less favorable lifestyle profiles.

## 2. Materials and Methods

### 2.1. Study Design and Population

**Figure 1:**
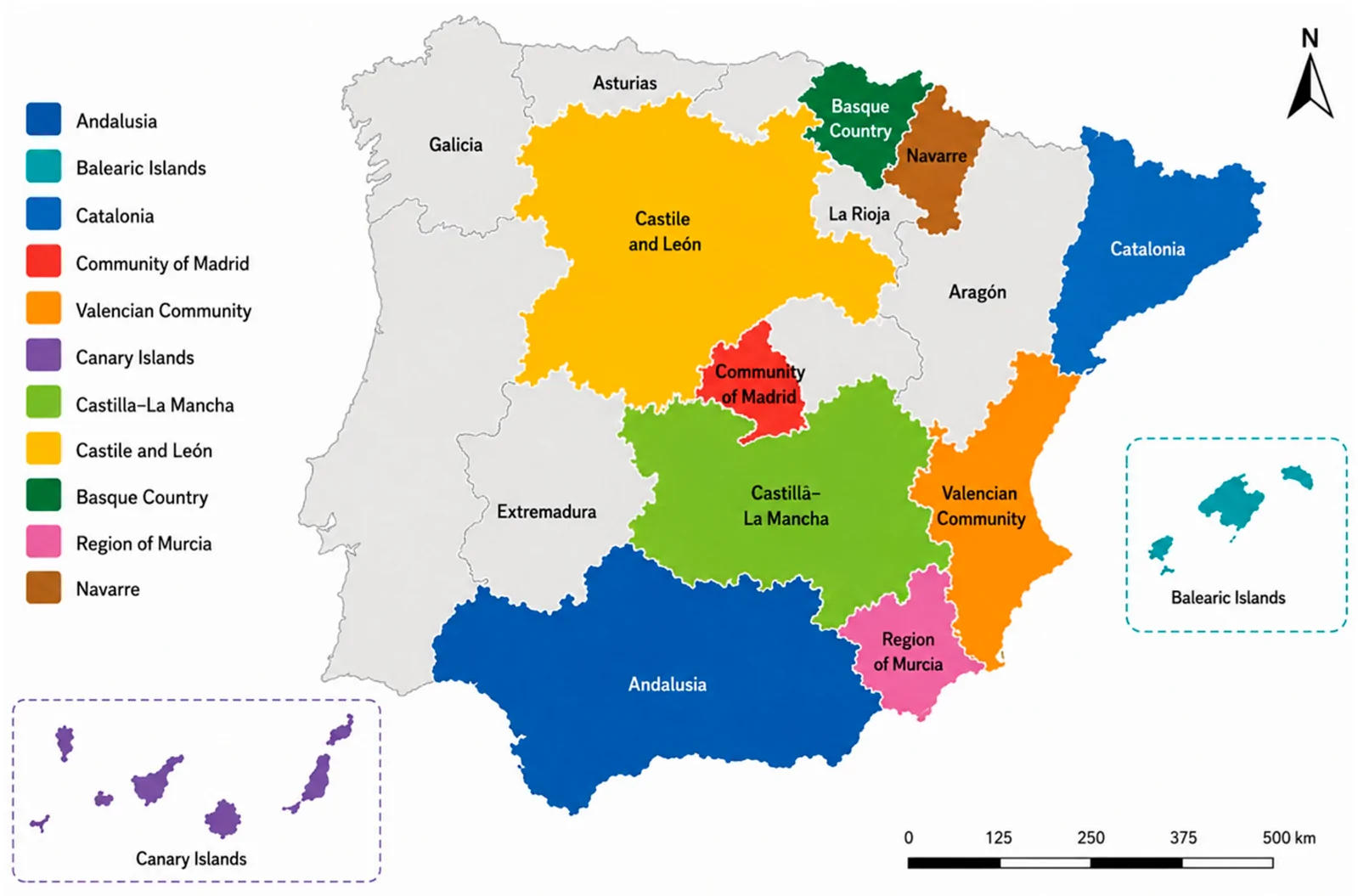
Geographical distribution of the Spanish autonomous communities included in the study.

A cross-sectional study was conducted using data obtained from routine occupational health examinations performed between January 2021 and December 2024 in ten Spanish autonomous communities: Andalusia, the Balearic Islands, Catalonia, the Community of Madrid, the Valencian Community, the Canary Islands, Castilla-La Mancha, the Basque Country, the Region of Murcia, Castile and León, and Navarre (Figure 1). These examinations were carried out by trained occupational health professionals as part of periodic workplace health surveillance programs established under Spanish occupational health regulations. Data collection followed standardized protocols that were applied uniformly across participating occupational health centers.

Initially, 105,472 actively employed workers participated in the health assessment process. Participants with incomplete information regarding technostress, lifestyle variables, or sociodemographic characteristics were excluded from the analysis. After applying the exclusion criteria, the final study population consisted of 104,175 workers.

The study was designed and reported according to the Strengthening the Reporting of Observational Studies in Epidemiology (STROBE) guidelines for observational research [23].

### 2.2. Inclusion and Exclusion Criteria

Eligible participants were men and women aged between 18 and 69 years who underwent a routine occupational health examination during the study period and completed all questionnaires included in the assessment protocol.

Participants were excluded if they presented:

- Incomplete Technostress Short Questionnaire (TCS-Short) data;
- Missing information regarding physical activity, Mediterranean diet adherence, or smoking status;
- Incomplete sociodemographic information;
- Duplicate records or inconsistent database entries.

**Figure 2:**
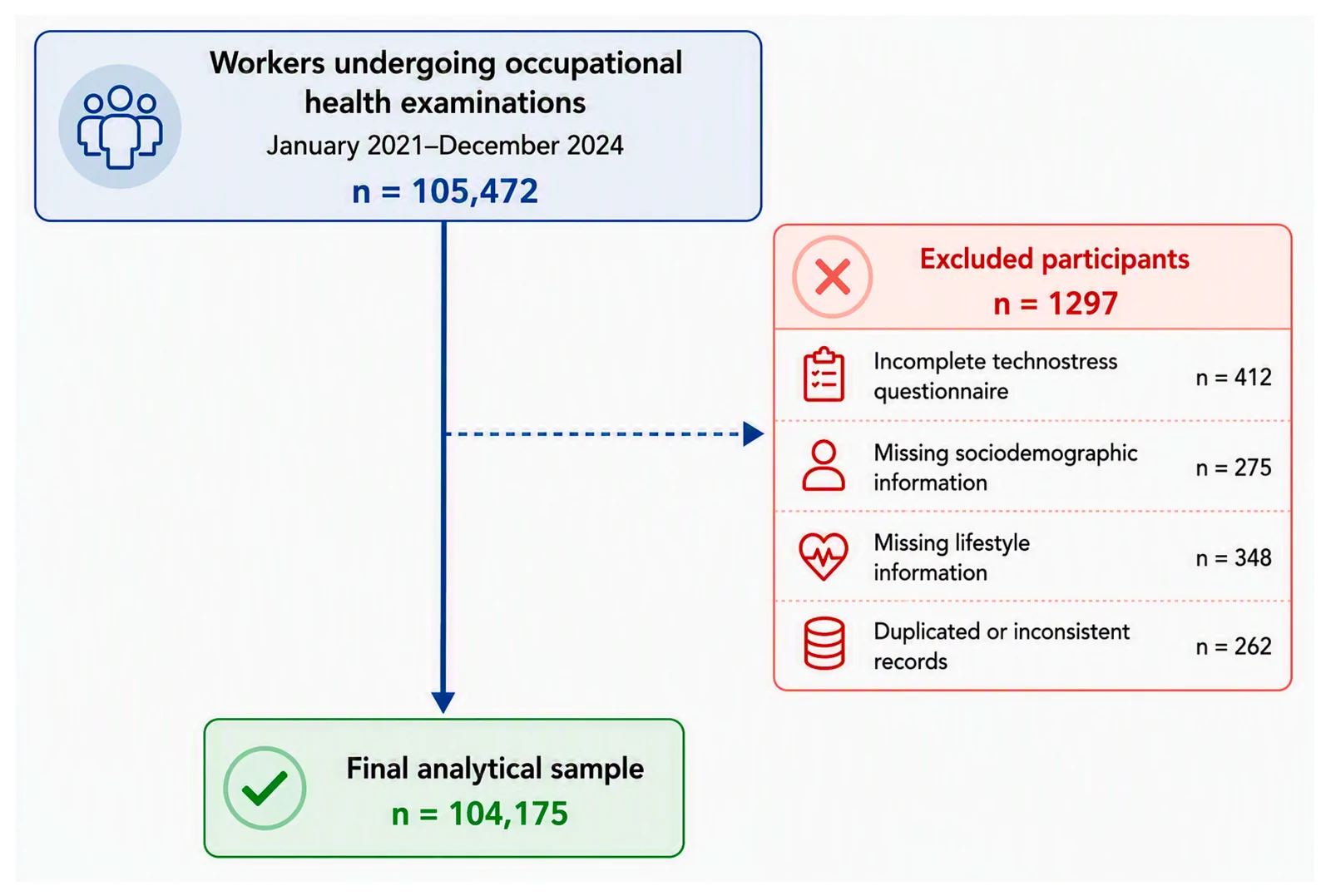
Flowchart of participant recruitment and selection. A total of 105,472 workers underwent routine occupational health examinations between January 2021 and December 2024. After exclusion of participants with missing or inconsistent information, 104,175 workers were included in the final analysis. Participants were classified according to their Healthy Lifestyle Score (HLS), calculated from physical activity, Mediterranean diet adherence, and smoking status, and subsequently evaluated for technostress levels.

A flow diagram describing participant selection is presented in Figure 2.

### 2.3. Sociodemographic Variables

Information regarding sex, age, educational attainment, and social class was obtained through standardized questionnaires administered during occupational health examinations.

Sex was classified as male or female.

Age was analyzed both as a continuous variable and as a categorical variable (<30, 30–39, 40–49, 50–59, and ≥60 years).

Educational attainment was categorized into three levels:

- Primary education;
- Secondary education;
- University education.

Social class was assigned according to occupational category using the classification proposed by the Spanish Society of Epidemiology based on the National Classification of Occupations (CNO-11), which groups workers according to occupational qualification, responsibility level, and employment status [24].

### 2.4. Lifestyle Variables

#### 2.4.1. Physical Activity

Physical activity was assessed using the short version of the International Physical Activity Questionnaire (IPAQ-SF) [25]. Weekly physical activity was expressed as metabolic equivalent minutes per week (MET-min/week) according to the IPAQ scoring protocol. MET-min/week were calculated by multiplying the reported minutes per day by the number of days per week and by the corresponding standard MET value for each activity intensity: 3.3 METs for walking, 4.0 METs for moderate-intensity activity, and 8.0 METs for vigorous-intensity activity. Total physical activity was calculated as the sum of walking, moderate-intensity, and vigorous-intensity MET-min/week. For construction of the Healthy Lifestyle Score, participants accumulating ≥600 MET-min/week were classified as physically active, whereas those accumulating <600 MET-min/week were classified as physically inactive. This threshold was selected to represent the minimum total weekly energy expenditure corresponding to at least a moderate level of physical activity according to the IPAQ scoring framework.

#### 2.4.2. Mediterranean Diet Adherence

Adherence to the Mediterranean diet was evaluated using the Mediterranean Diet Adherence Screener (MEDAS), a validated 14-item questionnaire originally developed within the PREDIMED study [26].

The questionnaire evaluates the consumption frequency of key components of the Mediterranean dietary pattern, including olive oil, vegetables, fruits, legumes, fish, nuts, and other characteristic food groups. Scores range from 0 to 14 points.

Consistent with previous studies, participants with scores ≥ 9 were classified as having high adherence to the Mediterranean diet [27].

#### 2.4.3. Smoking Status

Smoking habit was assessed through self-report. Participants who reported current tobacco consumption at the time of the examination were classified as smokers, whereas all remaining participants were considered non-smokers.

### 2.5. Construction of the Healthy Lifestyle Score

A Healthy Lifestyle Score (HLS) was created to evaluate the combined association of multiple health-related behaviors with technostress. Similar approaches have been widely used in studies investigating cardiometabolic health, mortality, and chronic disease risk [28,29,30].

One point was assigned for each of the following healthy behaviors:

- Regular physical activity (1 point);
- High adherence to the Mediterranean diet (1 point);
- Non-smoking status (1 point).

The resulting score ranged from 0 to 3 points, with higher values indicating a healthier lifestyle profile. The HLS was constructed as an unweighted behavioral count, with one point assigned to each health-promoting behavior. Equal weighting was used to provide a simple summary of the number of healthy behaviors present and should not be interpreted as implying equivalent associations of the individual components with technostress.

Participants were subsequently classified into four categories according to their total score:

- HLS = 0;
- HLS = 1;
- HLS = 2;
- HLS = 3.

### 2.6. Assessment of Technostress

Technostress was assessed using a Spanish-language 15-item short questionnaire covering five commonly described dimensions of technology-related occupational stress: techno-overload, techno-invasion, techno-complexity, techno-insecurity, and techno-uncertainty [31]. The questionnaire was administered directly in Spanish, and no translation or back-translation procedure was performed as part of the present study. Each dimension was represented by three items, rated on a five-point Likert scale ranging from 1 (strongly disagree) to 5 (strongly agree). An overall technostress score was calculated as the arithmetic mean of the 15 items. For descriptive purposes, the score was categorized using the operational intervals applied in the occupational-health assessment protocol: low (1.0–2.0), moderate (2.1–3.0), high (3.1–4.0), and very high (4.1–5.0). These categories should be regarded as operational categories rather than externally validated clinical cut-offs. For regression analyses, high and very high categories were combined to define the binary outcome (high-to-very high technostress), whereas low and moderate categories constituted the reference outcome. Only the resulting technostress classification was retained in the analytical dataset; item-level responses were not available for the present analysis. Consequently, internal-consistency coefficients, such as Cronbach’s alpha or McDonald’s omega, could not be estimated in this sample.

### 2.7. Statistical Analysis

Continuous variables are presented as means and standard deviations (SDs), whereas categorical variables are expressed as frequencies and percentages.

Differences in participant characteristics according to Healthy Lifestyle Score categories were assessed using one-way analysis of variance (ANOVA) for continuous variables and chi-square tests for categorical variables.

The prevalence of high technostress was calculated for each Healthy Lifestyle Score category. Trends across increasing HLS categories were evaluated using the chi-square test for trend.

Multivariable logistic regression models were constructed to estimate odds ratios (ORs) and 95% confidence intervals (95% CIs) for high technostress according to Healthy Lifestyle Score categories. Participants with HLS = 0 were used as the reference group.

The models were adjusted for:

- Sex;
- Age group;
- Educational level;
- Social class.

To examine the independent associations of the individual HLS components with high-to-very high technostress, an additional multivariable logistic regression model simultaneously included regular physical activity, high adherence to the Mediterranean diet, and non-smoking status, with adjustment for sex, age, educational level, and social class. In addition, the eight possible combinations of the three binary lifestyle behaviors were examined descriptively, and the number of participants and prevalence of high-to-very high technostress were calculated for each combination.

The primary analysis treated the Healthy Lifestyle Score as a categorical variable, with HLS = 0 as the reference category. An additional model treating HLS as an ordinal variable was initially explored; however, the categorical estimates were examined to assess whether the assumption of a progressive linear trend across HLS categories was supported.

Given the relatively high prevalence of high-to-very high technostress in some HLS categories, a sensitivity analysis was performed using Poisson regression with robust variance estimation to obtain adjusted prevalence ratios (aPRs) and 95% confidence intervals. The model used HLS = 0 as the reference category and included the same covariates as the primary logistic regression model (sex, age group, educational level, and social class).

Given the magnitude and precision of the associations observed for individual lifestyle components, variable coding, outcome coding, reference categories, and model specifications were re-examined. The analyses were repeated after verification of these parameters to ensure that the reported estimates reflected the intended comparisons.

Model discrimination was assessed using the area under the receiver operating characteristic curve (AUC), whereas calibration was evaluated using the Hosmer–Lemeshow goodness-of-fit test [32].

All analyses were performed using IBM SPSS Statistics version 30.0 (IBM Corp., Armonk, NY, USA). Statistical significance was established at *p* < 0.05.

### 2.8. Ethical Considerations

The study complied with the ethical principles outlined in the Declaration of Helsinki and with current European and Spanish legislation governing biomedical research and personal data protection [33,34].

All participants provided written informed consent before inclusion. The study protocol was approved by the Research Ethics Committee of the Balearic Islands (CEI-IB; reference IB 4383/20; approval date: 26 November 2020).

Data were anonymized before analysis, and only aggregated results are presented in this manuscript.

## 3. Results

### 3.1. Distribution of the Healthy Lifestyle Score

A total of 104,175 workers w included in the analysis. The Healthy Lifestyle Score (HLS) ranged from 0 to 3 points according to the presence of three healthy behaviors: regular physical activity, high adherence to the Mediterranean diet, and non-smoking status.

**Figure 3:**
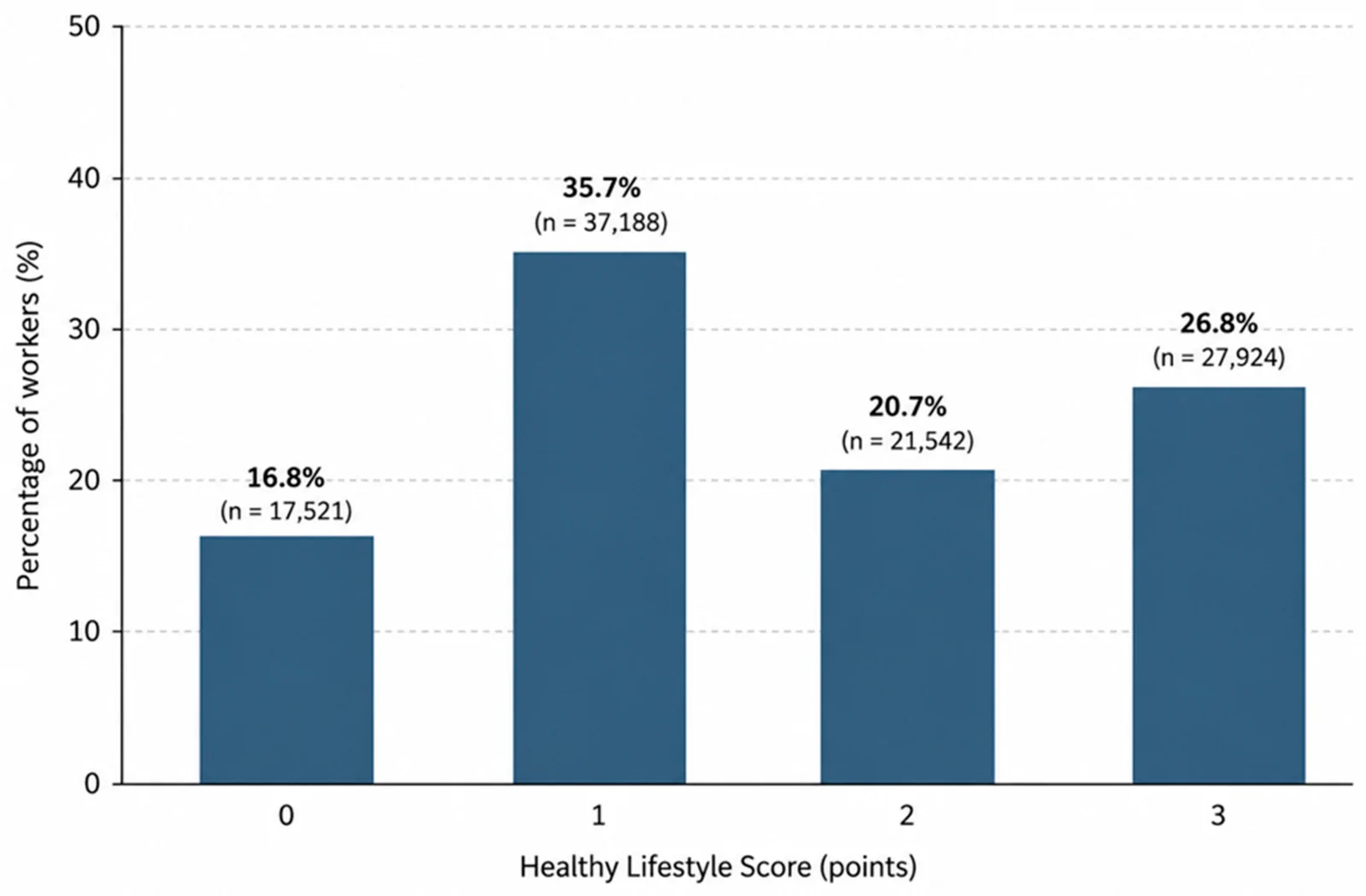
Distribution of the Healthy Lifestyle Score among Spanish workers.

Figure 3 shows the distribution of the Healthy Lifestyle Score in the study population. The largest proportion of workers was classified as HLS = 1 (35.7%), while 26.8% achieved the maximum score of 3 points. In contrast, only 16.8% of participants were categorized as HLS = 0, suggesting a relatively high prevalence of healthy lifestyle behaviors within this occupational cross-sectional sample.

### 3.2. Sociodemographic Characteristics According to Healthy Lifestyle Score

The sociodemographic characteristics of the study population according to Healthy Lifestyle Score categories are presented in Table 1. Significant differences were observed across all HLS categories (all *p* < 0.001). Workers with healthier lifestyle profiles were more frequently women, younger, had higher educational attainment, and were more likely to belong to higher social classes. The proportion of women increased from 30.3% in the HLS = 0 group to 44.9% in the HLS = 3 group, while the percentage of participants classified in social class I rose from 3.7% to 8.6%. Conversely, the proportion of workers in social class III decreased from 78.8% among those with HLS = 0 to 60.7% among those with HLS = 3. (Table 1).

### 3.3. Technostress Levels According to Healthy Lifestyle Score

Low technostress was observed in only 6.2% of workers with HLS 0 and 8.1% of those with HLS 1, compared with 36.8% and 43.8% among workers with HLS 2 and HLS 3, respectively.

The distribution of technostress levels according to Healthy Lifestyle Score categories is presented in Table 2. Significant differences were observed across all HLS categories (*p* < 0.001). Workers with healthier lifestyle profiles exhibited substantially lower levels of technostress. The prevalence of high-to-very high technostress was 50.8% among participants with HLS = 0 and 50.7% among those with HLS = 1, but decreased markedly to 7.5% and 7.2% among workers with HLS = 2 and HLS = 3, respectively. Conversely, the proportion of participants reporting low technostress increased progressively from 6.2% in the HLS = 0 group to 43.8% in the HLS = 3 group. These findings indicate a threshold-like pattern, with a markedly lower prevalence of high-to-very high technostress among workers with HLS = 2 and HLS = 3 compared with those with HLS = 0 and HLS = 1.

**Figure 4:**
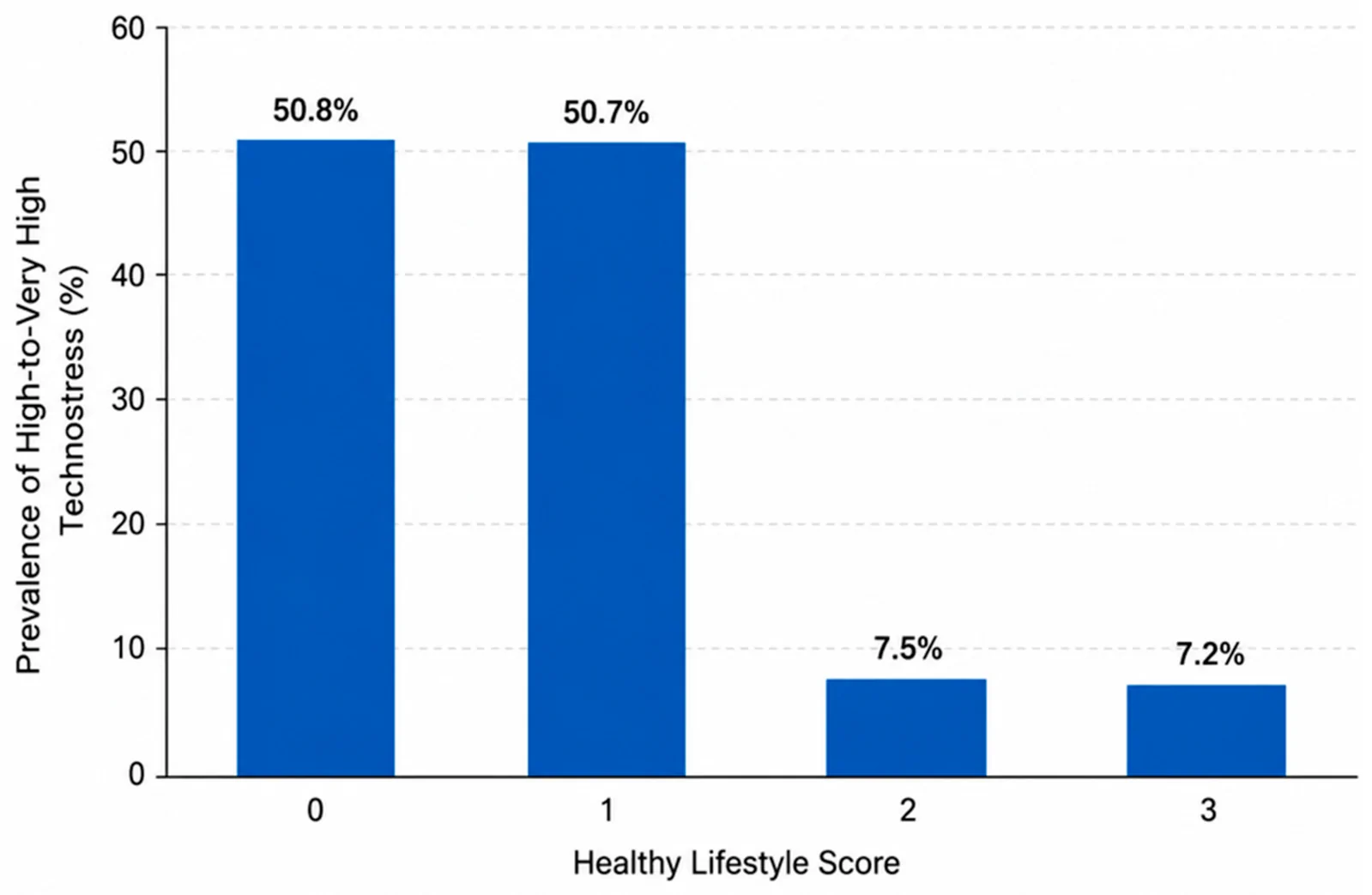
Prevalence of high-to-very high technostress according to Healthy Lifestyle Score.

Figure 4 illustrates the prevalence of high-to-very high technostress according to Healthy Lifestyle Score categories. The prevalence was similar among workers with HLS = 0 and HLS = 1 and markedly lower among those with HLS = 2 and HLS = 3, further supporting a threshold-like pattern. Additional component-specific analyses are presented in Supplementary Table S1. These models examined physical activity, Mediterranean diet adherence, and smoking status separately in relation to high-to-very high technostress, with adjustment for sex, age, educational level, and social class.

### 3.4. Association Between Healthy Lifestyle Score and High Technostress

The association between Healthy Lifestyle Score and high-to-very high technostress is presented in Table 3. In the crude analysis, workers with HLS values of 2 and 3 exhibited substantially lower odds of high technostress compared with those with HLS = 0. After adjustment for sex, age group, educational level, and social class, the association remained virtually unchanged. Compared with workers with HLS = 0, those with HLS = 2 had an adjusted OR of 0.073 (95% CI 0.068–0.078), while those with HLS = 3 had an adjusted OR of 0.074 (95% CI 0.069–0.079). In contrast, HLS = 1 was associated with slightly higher odds of high technostress compared with HLS = 0 (adjusted OR = 1.056; 95% CI 1.011–1.102). The categorical estimates therefore indicated a threshold-like, non-linear pattern, with a marked decrease in the odds of high technostress at HLS = 2 and no additional decrease at HLS = 3.

In the sensitivity analysis using Poisson regression with robust variance estimation, the corresponding adjusted prevalence ratios were 1.014 (95% CI 0.998–1.031; *p* = 0.085) for HLS = 1, 0.211 (95% CI 0.202–0.221; *p* < 0.001) for HLS = 2, and 0.211 (95% CI 0.203–0.220; *p* < 0.001) for HLS = 3, compared with HLS = 0 (Supplementary Table S2). Thus, although the prevalence-ratio estimates were less extreme than the odds ratios, the sensitivity analysis showed the same threshold-like pattern, with substantially lower prevalence of high-to-very high technostress for HLS = 2 and HLS = 3 but not for HLS = 1.

In a complementary model including the three HLS components simultaneously, together with sociodemographic covariates, the associations differed substantially across individual behaviors. Regular physical activity was associated with lower odds of high-to-very high technostress (adjusted OR = 0.145, 95% CI 0.135–0.157), as was high adherence to the Mediterranean diet (adjusted OR = 0.438, 95% CI 0.406–0.473). In contrast, non-smoking status was not associated with lower odds in the mutually adjusted model (adjusted OR = 1.190, 95% CI 1.145–1.236). These findings indicate that the individual components contributed differently to the associations summarized by the unweighted HLS (Supplementary Table S3).

Because identical HLS values may represent different combinations of lifestyle behaviors, the eight possible combinations of physical activity, Mediterranean diet adherence, and smoking status were additionally examined. The prevalence of high-to-very high technostress varied substantially across these combinations, indicating that the composition of the HLS categories was relevant to the observed associations. In particular, combinations including physical activity and/or Mediterranean diet adherence generally showed substantially lower technostress prevalence than profiles characterized only by non-smoking status (Supplementary Table S4).

**Figure 5:**
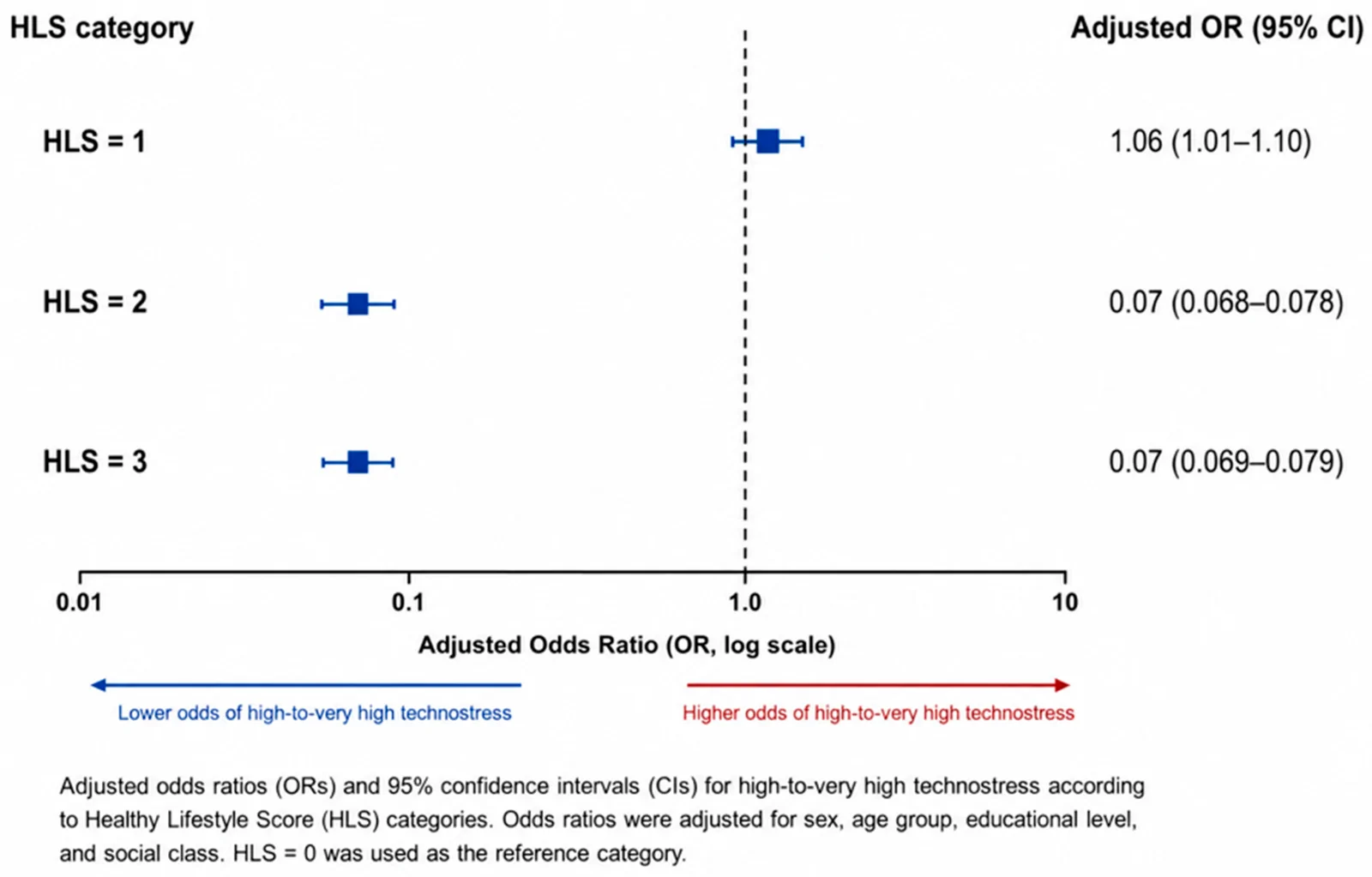
Adjusted odds ratios for high technostress according to Healthy Lifestyle Score.

Figure 5 presents the adjusted odds ratios for high-to-very high technostress according to Healthy Lifestyle Score categories. After adjustment for sex, age group, educational level, and social class, workers with HLS values of 2 and 3 exhibited markedly lower odds of high technostress compared with the reference category, whereas participants with HLS = 1 did not show lower odds of high technostress.

### 3.5. Model Performance

The performance of the fully adjusted logistic regression model was assessed by evaluating its discriminatory capacity. The model demonstrated excellent discrimination, with an area under the receiver operating characteristic curve (AUC) of 0.902, indicating a high ability to distinguish workers with high-to-very high technostress from those with low-to-moderate technostress.

Overall, the model showed high discrimination between workers with high-to-very high and low-to-moderate technostress. This model-performance result should be interpreted alongside the component-specific analyses, which showed substantial heterogeneity in the associations of the individual lifestyle behaviors with technostress.

## 4. Discussion

The present study examined the association between a combined Healthy Lifestyle Score (HLS) and technostress in a large cross-sectional sample of 104,175 Spanish workers. Three main findings emerged. First, substantial differences in technostress prevalence were observed according to lifestyle profile. Second, workers reporting two or more healthy lifestyle behaviors exhibited dramatically lower levels of high-to-very high technostress than those with less favorable behavioral profiles. Third, the categorical analysis revealed a threshold-like, non-linear association: HLS = 1 was not associated with lower odds of high technostress compared with HLS = 0, whereas HLS = 2 and HLS = 3 were associated with similarly and substantially lower odds. Collectively, these findings indicate a strong association between healthy lifestyle behaviors and technology-related occupational stress in increasingly digitalized work environments.

One of the most striking findings was the magnitude of the association observed between lifestyle profiles and technostress. While approximately half of workers with HLS values of 0 or 1 reported high-to-very high technostress, this prevalence decreased to approximately 7% among those achieving HLS values of 2 or 3. Similarly, multivariable analyses showed more than 90% lower odds of elevated technostress among workers with healthier lifestyle profiles. To our knowledge, few previous studies have evaluated the combined influence of multiple lifestyle behaviors on technostress, particularly in large occupational cohorts. Most available research has focused on organizational determinants such as workload, digital demands, role ambiguity, technological complexity, and organizational support. However, the present findings indicate that individual behavioral factors may also play a substantial role in shaping workers’ responses to technology-related stressors.

The observed association between healthier lifestyle profiles and lower technostress is biologically and psychologically plausible. Physical activity has consistently been associated with lower levels of perceived stress, anxiety, burnout, and psychological distress across occupational settings. Regular exercise modulates hypothalamic–pituitary–adrenal axis activity, improves autonomic nervous system balance, reduces systemic inflammation, and enhances emotional regulation and stress resilience. Recent evidence suggests that physically active individuals demonstrate greater adaptability to occupational stressors and recover more efficiently from psychological strain. These mechanisms may help explain why workers who engage in regular physical activity appear better equipped to cope with the cognitive and emotional demands associated with constant connectivity, information overload, and rapid technological change [35,36,37]. More broadly, lifestyle-related factors, including diet, physical activity, smoking, obesity, sleep, and psychological stress, have been linked to biological aging and stress-related health outcomes, further supporting the relevance of considering lifestyle patterns as interconnected determinants of health [38].

Adherence to the Mediterranean diet may represent an additional lifestyle factor associated with technostress. During the last decade, growing evidence has linked Mediterranean dietary patterns with improved mental health outcomes, lower depressive symptomatology, better cognitive performance, and enhanced psychological well-being. The anti-inflammatory and antioxidant properties of this dietary pattern, together with its effects on metabolic regulation and the gut–brain axis, may contribute to improved stress adaptation. Recent systematic reviews have demonstrated that higher adherence to Mediterranean dietary patterns is associated with lower levels of psychological distress and better overall mental health. Moreover, evidence from another Mediterranean population indicates that adherence to the Mediterranean diet may vary according to sociodemographic and contextual characteristics. In a Croatian study, higher adherence was associated with female sex, coastal residence, older age, higher educational attainment and income, and lower BMI [39]. These findings highlight the potential influence of sociodemographic and cultural context when interpreting associations involving Mediterranean diet adherence. Although specific evidence linking Mediterranean diet adherence to technostress remains scarce, the present findings suggest that nutritional factors may be relevant determinants of workers’ ability to cope with digital occupational demands [40,41,42].

Smoking behavior also deserves consideration. Although smoking has traditionally been perceived by some individuals as a mechanism for stress relief, contemporary evidence indicates that smokers frequently experience higher levels of anxiety, perceived stress, and psychological vulnerability than non-smokers. Nicotine dependence may generate cyclical fluctuations in mood and physiological activation that ultimately reinforce stress-related symptoms. However, the present component-specific analyses did not reproduce this expected direction for smoking status: after verification of the coding and reference categories, non-smoking was not associated with lower odds of high-to-very high technostress. In the present study, the mutually adjusted component-specific analysis showed substantial heterogeneity across the three behaviors included in the HLS. Regular physical activity and high Mediterranean diet adherence were associated with markedly lower odds of high-to-very high technostress, whereas non-smoking status was not associated with lower odds after simultaneous adjustment for the other lifestyle components and sociodemographic variables. These findings indicate that the components of the HLS should not be interpreted as having equivalent associations with technostress and support interpreting the HLS as an unweighted behavioral count rather than as a weighted risk index [43,44].

An important contribution of this study is the identification of a clear threshold pattern. Workers with HLS = 1 showed levels of technostress comparable to those observed among participants with HLS = 0, whereas substantially lower levels of technostress were observed among workers achieving HLS values of 2 or 3. However, the analysis of the eight possible behavior combinations showed that the composition of each HLS category is important for interpretation. Profiles characterized by physical activity and/or Mediterranean diet adherence generally showed substantially lower technostress prevalence, whereas non-smoking status alone did not show the same pattern. Thus, the threshold-like association observed for the composite HLS appears to reflect differences in the specific behaviors comprising each score category rather than an equivalent contribution of each additional healthy behavior. These findings are consistent with the growing literature on lifestyle clustering, which indicates that health-related behaviors frequently co-occur and that the composition of combined lifestyle profiles may be relevant to physical and mental health outcomes. Previous studies have shown that combined lifestyle scores are stronger predictors of cardiovascular disease, type 2 diabetes, metabolic syndrome, mental health outcomes, and mortality than individual behaviors considered separately [45,46,47].

The categorical analysis indicates that the association between HLS and technostress is better characterized as threshold-like and non-linear rather than as a progressive dose–response relationship. HLS = 1 was not associated with lower odds of high-to-very high technostress compared with HLS = 0, whereas HLS = 2 and HLS = 3 showed similarly and substantially lower odds. Accordingly, the categorical analysis was considered the primary basis for interpretation of the association.

From an occupational health perspective, these findings may have relevant practical implications. Most interventions designed to address technostress focus primarily on technological training, digital competencies, organizational support, or workload management. While such approaches are undoubtedly important, the associations observed in the present study suggest that lifestyle-related factors may also be relevant when considering workers’ susceptibility to technostress. Workplace programs encouraging regular physical activity, healthy dietary habits, smoking cessation, and overall well-being may therefore warrant further investigation in relation to workers’ adaptation to increasingly digitalized work environments. This perspective aligns with contemporary models of Total Worker Health, which emphasize the integration of occupational safety and health promotion strategies [48,49].

The study possesses several important strengths. First, the exceptionally large sample size provides substantial statistical power and enhances the precision of the estimates. Second, participants were recruited through routine occupational health examinations, increasing the relevance of the findings for real-world occupational settings. Third, validated instruments were used to assess physical activity, Mediterranean diet adherence, and technostress. Fourth, the construction of a combined lifestyle score allowed evaluation of cumulative behavioral effects rather than isolated exposures. Finally, the consistency of the findings across descriptive and multivariable analyses strengthens confidence in the robustness of the observed associations.

Several limitations should also be acknowledged. First, the cross-sectional design precludes establishing temporality or causal relationships between lifestyle behaviors and technostress. Reverse causality cannot be ruled out, as workers experiencing higher levels of technostress may have less time, motivation, or psychological resources to engage in regular physical activity or maintain healthy dietary habits. Therefore, the observed associations should not be interpreted as evidence that healthy lifestyle behaviors reduce technostress. Prospective longitudinal studies are needed to clarify the temporal direction of these relationships. Lifestyle variables were assessed through self-report questionnaires and may therefore be subject to recall and social desirability biases. In particular, participants may have overestimated socially desirable behaviors, such as physical activity or adherence to a healthy dietary pattern, while underreporting behaviors perceived as unhealthy, particularly tobacco consumption. This potential misclassification may have influenced the observed associations between the Healthy Lifestyle Score and technostress. In addition, both the lifestyle exposures and technostress outcome were assessed using self-reported information collected within the same assessment context, raising the possibility of common-method bias. Correlated reporting tendencies or other shared measurement-related factors could therefore have contributed to the observed associations. A further limitation concerns the construction of the HLS as an equally weighted score. Although this approach provides a simple summary of the number of healthy behaviors, the component-specific analyses showed markedly different associations with technostress across physical activity, Mediterranean diet adherence, and smoking status. Moreover, the analysis of the eight possible behavior combinations showed that participants with the same HLS value could have different behavioral profiles and technostress prevalence. Accordingly, the HLS should be interpreted as an unweighted behavioral count rather than as a measure in which each component contributes equally to the observed association. In addition, information on passive smoking exposure and alcohol consumption was not collected. Consequently, these potentially relevant lifestyle factors could not be evaluated in relation to technostress or considered in the construction of the Healthy Lifestyle Score. Additionally, detailed information on the digital work context was not available, including the intensity and duration of technology use (e.g., daily screen time), the types of digital tools or software used, constant availability or connectivity through smartphones, remote working arrangements, organizational support, and digital competence. These technology-specific factors are relevant determinants of technostress, and their absence limits our ability to characterize workers’ exposure to digital demands and to determine the extent to which differences in the digital work environment may account for the observed associations. Residual confounding by unmeasured occupational and contextual factors cannot therefore be excluded. In addition to the digital-work characteristics described above, information on working hours, specific job demands, calendar year of assessment, and identifiers for employer, occupational-health center, or region was not available in the analytical dataset. Consequently, these factors could not be incorporated into the multivariable models. The absence of employer-, center-, or region-level identifiers also precluded the use of multilevel models or cluster-robust standard errors to account for potential within-cluster correlation. Although data collection followed standardized protocols across participating occupational health centers, unmeasured workplace or contextual characteristics may have influenced both lifestyle behaviors and technostress. The magnitude and precision of the reported associations should therefore be interpreted with appropriate caution. Furthermore, the study population consisted exclusively of actively employed workers undergoing occupational health surveillance, which may have introduced a healthy worker effect. Workers who had already left employment or were absent from work because of burnout, severe stress, or other stress-related health problems may therefore be underrepresented or absent from the study population. Consequently, the prevalence of high technostress observed in this sample may underestimate its prevalence in the broader working population. This selection mechanism may also have introduced selection bias if participation in occupational health surveillance or inclusion in the analytical sample was related to both lifestyle characteristics and technostress. Finally, the study population consisted exclusively of Spanish workers, which may limit the generalizability of our findings to other countries and cultural or occupational contexts. This consideration may be particularly relevant because adherence to the Mediterranean diet constitutes one of the three components of the Healthy Lifestyle Score and may reflect dietary patterns that are more prevalent or culturally embedded in Mediterranean populations. Therefore, the composition and distribution of the HLS, as well as its association with technostress, may differ in populations with substantially different dietary habits, lifestyle patterns, working conditions, or occupational cultures. Further studies in non-Spanish and non-Mediterranean working populations are needed to assess the external validity of these findings.

Future research should investigate whether interventions targeting multiple lifestyle behaviors can effectively reduce technostress over time. Prospective studies could help determine whether improvements in physical activity, dietary habits, and smoking cessation lead to measurable reductions in technology-related stress. Additionally, studies incorporating objective indicators of digital exposure, wearable technologies, sleep assessment, and biomarkers of stress may provide further insight into the biological pathways linking lifestyle behaviors and technostress. Understanding these mechanisms will become increasingly important as digital technologies continue to reshape modern workplaces.

Overall, the present findings indicate a strong association between lifestyle profiles and technostress, although this association was not uniform across the individual behaviors comprising the HLS. The categorical and behavioral-combination analyses suggest a threshold-like pattern that was largely associated with profiles including physical activity and/or Mediterranean diet adherence. These findings highlight the importance of considering both the number and the specific combination of lifestyle behaviors when interpreting the association between the HLS and technostress.

## 5. Conclusions

In this large cross-sectional study of 104,175 Spanish workers, a strong and consistent association was observed between healthy lifestyle behaviors and lower levels of technostress. Workers with HLS = 2 or HLS = 3 showed substantially lower prevalence and odds of high-to-very high technostress than those with HLS = 0 or HLS = 1; however, component-specific and behavioral-combination analyses indicated that these associations differed according to the specific behaviors comprising the score, with physical activity and Mediterranean diet adherence showing stronger associations than smoking status.

The categorical analysis revealed a threshold-like, non-linear association rather than a progressive dose–response relationship, with substantially lower odds observed for HLS = 2 and HLS = 3 but not for HLS = 1. Additional component-specific and behavioral-combination analyses showed that this pattern was not attributable to an equivalent contribution of each HLS component. Rather, the associations differed substantially according to the behaviors comprising the score, underscoring the importance of interpreting the HLS as an unweighted behavioral count.

These findings extend current knowledge on technostress by showing that, beyond organizational and technological factors, individual lifestyle characteristics are also associated with workers’ responses to increasingly digitalized work environments. The observed associations suggest that healthier lifestyle profiles are related to lower vulnerability to the cognitive and emotional demands associated with intensive ICT use.

From a practical perspective, the observed associations suggest that physical activity, dietary quality, and smoking status may be relevant lifestyle factors to consider alongside established organizational and technological determinants of technostress. However, intervention studies are needed to determine whether workplace health-promotion programs targeting these behaviors can lead to changes in technostress.

Although the cross-sectional design precludes causal inference, the strength and consistency of the observed associations support the relevance of lifestyle-related factors in the study of technostress. Longitudinal and intervention studies are warranted to establish temporal relationships and determine whether changes in lifestyle behaviors are followed by changes in technostress and adaptation to the rapidly evolving digital workplace.

## Figures and Tables

**Table 1:** Sociodemographic characteristics of the study population according to Healthy Lifestyle Score.

Variable	HLS = 0 (*n* = 17,521)	HLS = 1 (*n* = 37,188)	HLS = 2 (*n* = 21,542)	HLS = 3 (*n* = 27,924)	*p*-Value
Sex, *n* (%)					<0.001
Male	12,209 (69.7)	23,191 (62.4)	12,076 (56.1)	15,400 (55.1)	
Female	5312 (30.3)	13,997 (37.6)	9466 (43.9)	12,524 (44.9)	
Age (years), mean ± SD	41.5 ± 9.6	42.8 ± 10.2	36.1 ± 9.7	36.7 ± 9.5	<0.001
Educational level, *n* (%)					<0.001
Primary education	11,549 (65.9)	21,828 (58.7)	12,937 (60.1)	13,430 (48.1)	
Secondary education	5340 (30.5)	13,310 (35.8)	7466 (34.7)	12,173 (43.6)	
University education	632 (3.6)	2050 (5.5)	1139 (5.3)	2321 (8.3)	
Social class, *n* (%)					<0.001
Class I	642 (3.7)	2099 (5.6)	1149 (5.3)	2390 (8.6)	
Class II	3067 (17.5)	8234 (22.1)	4923 (22.9)	8577 (30.7)	
Class III	13,812 (78.8)	26,855 (72.2)	15,470 (71.8)	16,957 (60.7)	

Data are presented as mean ± standard deviation (SD) for continuous variables and as number (percentage) for categorical variables. *p*-values were obtained using one-way analysis of variance (ANOVA) for continuous variables and χ^2^ tests for categorical variables. HLS, Healthy Lifestyle Score; SD, standard deviation.

**Table 2:** Distribution of technostress levels according to Healthy Lifestyle Score.

Technostress Level	HLS = 0	HLS = 1	HLS = 2	HLS = 3	*p*-Value
Low	1084 (6.2)	3010 (8.1)	7934 (36.8)	12,230 (43.8)	<0.001
Moderate	7536 (43.0)	15,323 (41.2)	11,982 (55.6)	13,672 (49.0)	<0.001
High	6623 (37.8)	12,878 (34.6)	1505 (7.0)	1895 (6.8)	<0.001
Very High	2278 (13.0)	5977 (16.1)	121 (0.6)	127 (0.5)	<0.001
High + Very High	8901 (50.8)	18,855 (50.7)	1626 (7.5)	2022 (7.2)	<0.001

Distribution of technostress levels according to Healthy Lifestyle Score (HLS) categories. Data are presented as number (percentage). *p*-values were obtained using χ^2^ tests. HLS, Healthy Lifestyle Score.

**Table 3:** Crude and adjusted odds ratios for high-to-very high technostress according to Healthy Lifestyle Score.

Healthy Lifestyle Score	Crude OR (95% CI)	Adjusted OR * (95% CI)	*p*-Value
HLS = 0	Reference	Reference	—
HLS = 1	1.00 (0.96–1.03)	1.06 (1.01–1.10)	0.015
HLS = 2	0.08 (0.08–0.08)	0.07 (0.068–0.078)	<0.001
HLS = 3	0.08 (0.07–0.08)	0.07 (0.069–0.079)	<0.001

Crude and adjusted odds ratios (ORs) and 95% confidence intervals (CIs) for high-to-very high technostress according to Healthy Lifestyle Score (HLS) categories. Adjusted for sex, age group, educational level, and social class. * Adjusted odds ratios represent the association between Healthy Lifestyle Score (HLS) categories and high-to-very high technostress after controlling for potential confounders. HLS = 0 was used as the reference group.

## Data Availability

The datasets generated and/or analyzed during the current study are stored at ADEMA University School. Data are available from the corresponding author upon reasonable request and subject to compliance with applicable ethical, legal, and data protection requirements.

## Supplementary Materials

The following supporting information can be downloaded at: https://www.mdpi.com/article/10.3390/nu18172754/s1, Table S1: Association between individual healthy lifestyle behaviors and high technostress; Table S2: Sensitivity analysis of the association between Healthy Lifestyle Score and high-to-very high technostress using Poisson regression with robust variance estimation; Table S3: Mutually adjusted associations between individual Healthy Lifestyle Score components and high-to-very high technostress; Table S4: Distribution and prevalence of high-to-very high technostress according to the eight possible combinations of lifestyle behaviors.
