# Supplementary material for: Healthy Lifestyle Behaviors and Technostress: A Combined Lifestyle Score Analysis in 104,175 Spanish Workers
Source: Nutrients. 2026 Aug 23;18(17):2754. doi: 10.3390/nu18172754 (PMC13567263; doi:10.3390/nu18172754)
Supplement: Supplementary file 1 [file nutrients-18-02754-s001.zip › nutrients-4494301-supplementary.pdf]

Supplementary Table S1. Association between individual healthy lifestyle behaviors and high technostress.

Variable	Adjusted OR	95% CI
Physical activity	0.074	0.071–0.077
Mediterranean diet adherence	0.085	0.081–0.089
Current smoking	0.828	0.800–0.857

OR, odds ratio; CI, confidence interval. Each lifestyle behavior was examined in a separate multivariable logistic regression model adjusted for sex, age, educational level, and social class. The dependent variable was high-to-very high technostress (vs. low-to-moderate technostress). For smoking status, non-smoking was the reference category; therefore, the reported OR corresponds to current smoking versus non-smoking.

Supplementary Table S2. Sensitivity analysis of the association between Healthy Lifestyle Score and high-to-very high technostress using Poisson regression with robust variance estimation.

Healthy Lifestyle Score	Adjusted prevalence ratio (aPR)	95% CI	p-value
HLS = 0	1.000 (Reference)	—	—
HLS = 1	1.014	0.998–1.031	0.085
HLS = 2	0.211	0.202–0.221	<0.001
HLS = 3	0.211	0.203–0.220	<0.001

aPR, adjusted prevalence ratio; CI, confidence interval; HLS, Healthy Lifestyle Score. Estimates were obtained using Poisson regression with robust variance estimation and were adjusted for sex, age group, educational level, and social class. HLS = 0 was used as the reference category.

Supplementary Table S3. Mutually adjusted associations between individual Healthy Lifestyle Score components and high-to-very high technostress.

Lifestyle component	Reference category	Adjusted OR*	95% CI	p-value
Regular physical activity	Physical inactivity	0.145	0.135–0.157	<0.001
High Mediterranean diet adherence	Low/moderate adherence	0.438	0.406–0.473	<0.001
Non-smoking status	Current smoking	1.190	1.145–1.236	<0.001

*OR, odds ratio; CI, confidence interval; HLS, Healthy Lifestyle Score. All three lifestyle components were entered simultaneously into the same multivariable logistic regression model and additionally adjusted for sex, age, educational level, and social class. The dependent variable was high-to-very high technostress (vs. low-to-moderate technostress).

Supplementary Table S4. Distribution and prevalence of high-to-very high technostress according to the eight possible combinations of lifestyle behaviors.

Physical activity	Mediterranean diet adherence	Non-smoking	n	High-to-very high technostress, n (%)
No	No	No	17,521	8,901 (50.8%)
No	No	Yes	34,440	18,599 (54.0%)
No	Yes	No	769	43 (5.6%)
No	Yes	Yes	1,428	126 (8.8%)
Yes	No	No	1,979	216 (10.9%)
Yes	No	Yes	3,392	297 (8.8%)
Yes	Yes	No	16,722	1,203 (7.2%)
Yes	Yes	Yes	27,924	2,022 (7.2%)
